# The Damietta Server: a comprehensive protein design toolkit

**DOI:** 10.1093/nar/gkae297

**Published:** 2024-04-25

**Authors:** Iwan Grin, Kateryna Maksymenko, Tobias Wörtwein, Mohammad ElGamacy

**Affiliations:** Interfaculty Institute of Microbiology and Infection Medicine (IMIT), University of Tübingen, Tübingen, Germany; Max Planck Institute for Biology, Department of Protein Evolution, Tübingen, Germany; Max Planck Institute for Biology, Department of Protein Evolution, Tübingen, Germany; Division of Translational Oncology, Internal Medicine II, University Hospital Tübingen, Tübingen, Germany; Max Planck Institute for Biology, Department of Protein Evolution, Tübingen, Germany; Division of Translational Oncology, Internal Medicine II, University Hospital Tübingen, Tübingen, Germany

## Abstract

The growing importance of protein design to various research disciplines motivates the development of integrative computational platforms that enhance the accessibility and interoperability of different design tools. To this end, we describe a web-based toolkit that builds on the Damietta protein design engine, which deploys a tensorized energy calculation framework. The Damietta Server seamlessly integrates different design tools, in addition to other tools such as message-passing neural networks and molecular dynamics routines, allowing the user to perform multiple operations on structural models and forward them across tools. The toolkit can be used for tasks such as core or interface design, symmetric design, mutagenic scanning, or conformational sampling, through an intuitive user interface. With the envisioned integration of more tools, the Damietta Server will provide a central resource for protein design and analysis, benefiting basic and applied biomedical research communities. The toolkit is available with no login requirement through https://damietta.de/.

## Graphical Abstract

**Graphical Abstract:**
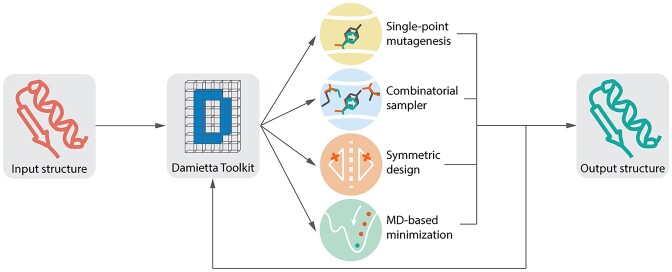

## Introduction

Owing to its rapid progress, *de novo* protein design has lent itself to becoming an established procedure for creating proteins with novel functions and applications (1–3). Furthermore, computational protein design is now increasingly guiding empirical engineering approaches, such as directed evolution, which not only leads to superior results, but also rationalizes the observed protein fitness (4). To those ends, physics-based methods (5) are indispensable for designing proteins to atomic-level details, provide for generalizable design frameworks, and are instrumental in elucidating sequence–structure relationships. Moreover, integrating these methods into specialized pipelines that incorporate evolutionary profiles (6,7), statistical potentials (8,9), geometric patterns (10,11) or deep learning models (12) is very effective in yielding functional proteins. However, the specialized expertise required for setting up physics-based methods and integrating them with other tools prohibit their broader use by the biochemistry, biotechnology and medical research communities.

**Figure 1.**
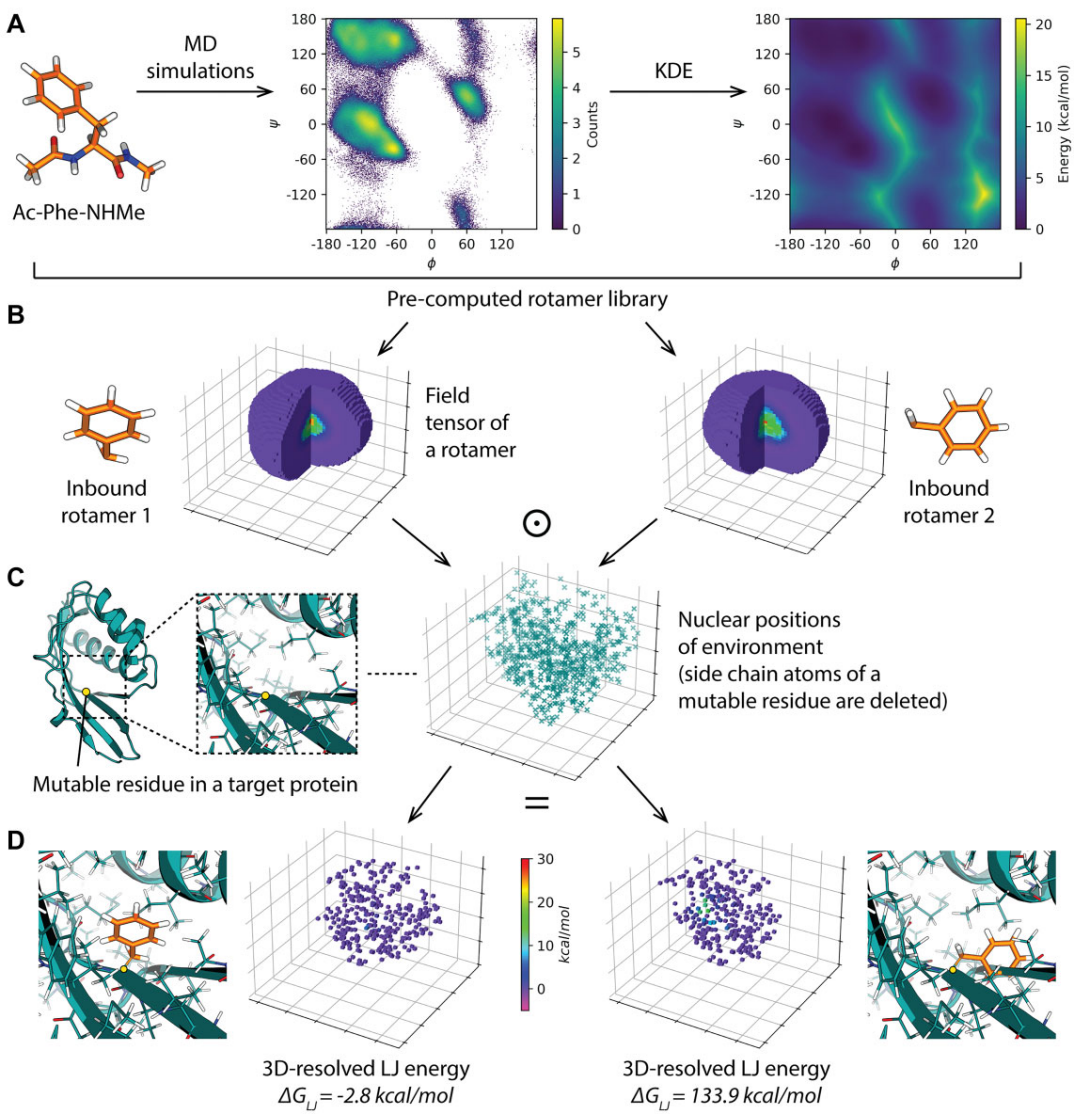
Overview of library generation and energy calculations in Damietta. (**A**) At the library generation stage, representative conformers are extracted from a pool of molecular dynamics simulations of capped-termini amino acids. Backbone energies are derived from a smoothed energy landscape by kernel density estimation, and side chain energies are derived from clusters of conformational distributions. (**B**) Conformers from a given range of ($\varphi ,\;\psi$) angles are stored in the rotamer library along with their precomputed rotamer field tensors. Shown are example conformations of phenylalanine and their associated Lennard-Jones field tensors. (C, D) The interaction energy is evaluated through multiplying the rotamers field tensors by a 3D histogram of the protein environment atomic positions (**C**). This yields the energy values that recapitulate a favorable (left) or a high-energy (right) interaction (**D**).

Recently, we have developed Damietta, a framework that introduces a new approach to protein design computations (13). Unlike previous methods which evaluate interaction energies by looping over all interacting atom pairs, our framework calculates the interaction energies between two groups of atoms through a single tensor operation. Applied to design, this *single instruction, multiple data* scheme can evaluate a much larger number of rotamers at atomistic resolution than existing methods. Furthermore, most of the interaction information is precomputed and stored for the rotamer library as 3D projections, vastly reducing runtime. Finally, this 3D tensorization results in grids with constant dimensions, facilitating multi-level parallelization (Figure 1). The Damietta protein design framework is based on an energy function, molecular parameters, and a rotamer library derived from a single molecular mechanics force field. This has the two-fold advantage of relying on self-consistent scoring terms, and avoiding any training procedure for parametrizing them. Using the Damietta design software, we successfully designed copper-binding and EGFR-inhibiting proteins, and could improve the affinity and tune the activity of G-CSFR-binding designs (13,14).

Herein we report the Damietta Server, which we created with the aim of integrating several protein design and modeling tools on one platform with a user-friendly graphical interface. Specifically, we implement three Damietta applications for combinatorial (cs) or symmetric (sd) design and forced mutagenesis (sp) tasks. In these implementations, we further combine the Damietta discrete rotamer samplers with molecular dynamics minimization routines to achieve full flexibility and improve the quality of the design models. Moreover, the Damietta Server is also equipped with additional third-party tools to help users with assessing the input and output of design tasks. Specifically, in this version of the server, we implement message-passing neural networks (15) to generate a position-specific mutation probability matrix. Additionally, we deploy molecular dynamics tools (16) for energy minimization and quality assessment of the resulting design models. Finally, with inter-operability in mind, the Damietta Server was built to follow structure-centric flow, where the input and output structure models can be forwarded seamlessly across the different tools. These features position the Damietta Server as a comprehensive design and modelling platform for a wide range of tasks.

## Results

### Overview of the server workflow, input and output

**Figure 2.**
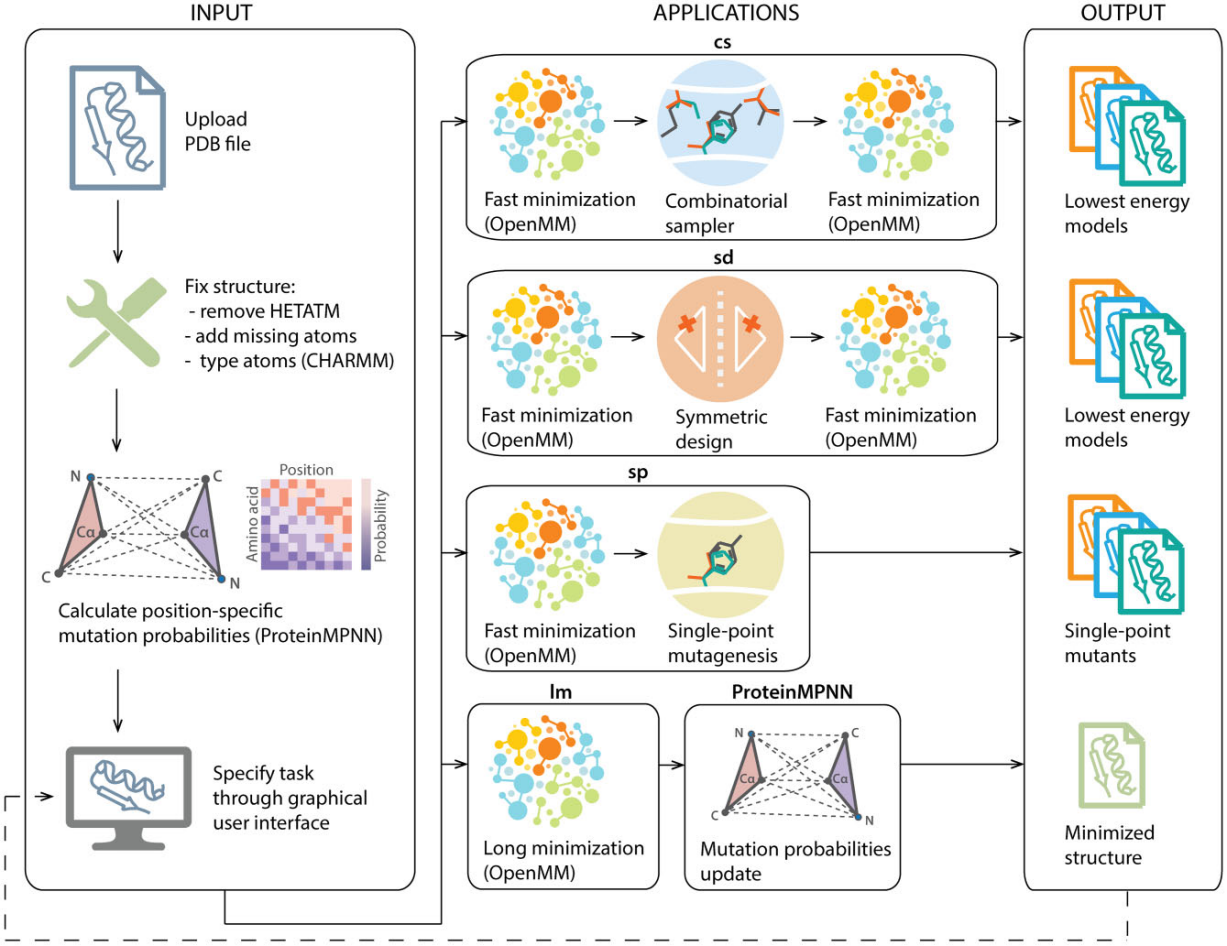
Overview of the Damietta Server workflows. In the pre-processing stage of the user-input structure (left pane): (i) all heteroatoms are removed, (ii) missing hydrogens or heavy atoms are added and (iii) atom types are assigned according to the used force field. Next, a position-specific mutation probability matrix is generated for the input backbone coordinates using ProteinMPNN(15). Finally, the pre-processed structure and its sequence and mutation probabilities are graphically displayed, where the user can choose a simulation type and its parameters. The available applications (middle pane) allow the user to perform: (i) flexible-backbone design (***cs***) or repacking of multiple residues, (ii) flexible-backbone design with symmetry constraints (***sd***), (iii) modeling single-point mutants (***sp***), or long minimization by molecular dynamics (***lm***) using OpenMM(16). The output structures and sequences (right pane) are then graphically displayed, where the user can select a model and forward it for further simulations.

The Damietta Sever deploys a three-stage workflow for all operations. The workflow starts and ends by a structure, whereby the output structure can be further forwarded as input for a new operation (Figure 2). At the first stage of the workflow, the structure is rapidly pre-processed, analyzed and displayed. The pre-processing involves the removal of all non-protein atoms, adding hydrogens and other missing side chain atoms, and assigning the type of each atom according to the CHARMM36 force field (17). Next, the backbone structure is analyzed for the mutational probabilities at every position as predicted by ProteinMPNN (15). This yields two position-specific mutations probability matrices that remain associated with the structure throughout the workflow. The two probability matrices are derived from standard and solubility-enhancing neural network weights. At the end of the first stage, the pre-processed input structure and sequence are graphically displayed, with input fields to specify the simulation type and parameters.

The Damietta design engine executes one of two core operations to any selected amino acid position; *repacking*, or *mutating* the existing side chain. In either case, the side chain atoms of a target position are deleted, leaving behind the atoms that describe the host environment of an inbound rotamer. In a repacking instance, different conformations of the wild-type side chain are evaluated for their internal and interaction energies with the host protein environment, and the lowest energy conformer is kept. The same procedure is performed for mutagenesis, which additionally evaluates side chain conformations of all target amino acid types. The Damietta design tools perform repacking and mutagenesis operations in various sampling orders, according to the intended application. All the tools however output a set of lowest-energy models, along with a per-residue breakdown for each energy term of the Damietta energy function. Finally, the long minimization tool performs all-atom minimization dynamics and outputs a single model representing the final frame of the simulation. The user can start multiple runs with different tools and parameters in parallel. To iteratively refine models, the output models can directly be used as inputs for subsequent runs within the Damietta protein design toolkit.

### Design algorithms and associated tools

The Damietta energy function is composed of five additive terms representing residue-wise internal energies and residue-environment interaction energies. The internal energies terms, denoted $\Delta {G_{pp}}$ and $\Delta {G_k}$, are derived during the generation of the rotamer library and represent the backbone torsions and side chain conformational distributions, respectively. In the current version of the Damietta software (v1.60), the library is pre-generated using molecular dynamics trajectories of capped amino acids. The resulting conformational ensemble is used to construct the $( {\varphi ,\psi } )$ distribution for each amino acid, which is in turn used to derive a probability density function using periodic kernel density estimation (Figure 1A). The latter probability density map is looked up to identify the energy $\Delta {G_{pp}}{\mathrm{(}}{X_i}{\mathrm{|}}{\varphi _i},{\psi _i})$ of placing amino acid $X$ at position $i$. The conformational pool is gridded into a total of 36 ($\varphi ,\;\psi$) bins, whereby side chain conformations are $k$-means clustered within each bin to derive side chain energy values $\Delta {G_k}$. $\Delta {G_k}$ represents the Boltzmann weight of the relative cluster sizes for each of the $k$ representative conformations from each cluster. The Lennard-Jones, electrostatic, and solvation *field tensors* are pre-computed for this constant number of rotamers ($k = 100)$ for each ($\varphi ,\;\psi$) range. During runtime, for a given amino acid position, the side chain atoms are removed and grid environment is mapped into a 3D histogram of the surrounding atoms. This *environment tensor* is then multiplied by the rotamer field to yield the position-resolved interaction energies (Figure 1C, D). The sum of these energy terms yields the total energy $\Delta {G_{total}}$ at a target position. Hence, all the Damietta tools that handle (i.e. repack or mutate) more than one amino acid position additionally report the average per-residue energy, this provides a comparable metric across proteins of different sizes (13). The following section details the various operations the user can apply to input model:

1. ***Side chain repacking*.** Absent any specified mutable positions, the user can use the interface of any Damietta tool (i.e. ***cs***, ***sd***, or ***sp*** interfaces) to sample the side chain conformations of selected repackable residues, and report their average per-residue energies. This repacks the specified residues deterministically in their input order. The input and output structure will both undergo fast all-atom minimization, leading to lower-energy models. This tool is useful for local evaluation of energy for selected residues or for quick relaxation of the entire structure.
2. ***Single-point mutagenesis (sp) tool*.** The user can evaluate the isolated impact of individual mutations with or without repacking of additional residues. The single-point mutagenesis protocol forces each of the specified mutations individually by evaluating 100 side chain conformers for each of the specified target mutations. The resulting models minimize the side chain energies over a single mutable position and all selected repackable positions in a model. This is useful for reporting energies even of detrimental (i.e. energy-raising) mutations. To cover the scenarios in which energy is raised due to a mutation, the protocol first evaluates the target mutation, and if it results in a steric clash, the maximally-permitted LJ ceiling (i.e. max_lj parameter) is raised to accommodate sub-optimal rotamer placement (Supplementary methods). Hence, no all-atom minimization cycle is performed on the resulting model. The user can also use the sp tool to rapidly model a large number of single-point mutant structures in a single run. Similar to the other Damietta tools, the average per-residue and per-term energy breakdowns are reported.
3. ***Combinatorial sampler (cs) tool*.** Given the dimensionality of the combinatorial design space of more than a few amino acid positions, the outcomes are largely influenced by the sampling algorithm. In such design spaces, both excessive entrapment or stochasticity can derail the sampling process from efficient minimization. Hence, we implement a swarm algorithm that tracks multiple, loosely-communicating search paths, starting from a randomized order of the mutagenesis decision tree. The algorithm deploys exploratory mutagenesis and extensive repacking routines to identify successively lower local minima, the swarms are respawned from the lowest identified minima of a few unique sequences (i.e. n_paths; Supplementary methods). This algorithm provides a balance between conservative minimization and stochastic diversity. The results represent the lowest-energy, unique-sequence models at the end of each swarm path. Although the core of this tool performs the combinatorial search in a fixed-backbone context, a preceding and a subsequent cycle of short molecular dynamics minimization are applied. This introduces all-atom motions (including the backbone) for each designed sequence (Figure 2) and introduces further conformational diversification the resulting models, especially with repeated cycles of design and minimization.
4. ***Symmetric design (sd) tool*.** Through this tool the user can explicitly specify symmetric design constraints, which will sample the same mutations over multiple specified positions, simultaneously. While the user can input design templates that comprise any level of structural symmetry, symmetric design in Damietta enforces symmetry only on the sequence level. Avoiding symmetry on the conformation level of rotamer placement leads to more rigorous sampling of the energy landscape and better physical relevance of the resulting model. The symmetric design protocol follows a similar flow control to the combinatorial sampler (cs) with the addition of an inner symmetric mutagenesis loops that attempts the symmetric mutations in the provided order of symmetric positions (Supplementary methods section). Similarly, the resulting models are relaxed by fast molecular dynamics minimization before and after the design stage (Figure 2).
5. ***Long minimization (lm) tool*.** In order to provide a generic, continuous conformational sampling tool, we set up a molecular mechanics routine to carry out an all-atom minimization for any input model. The tool uses the CHARMM36 force field and yields the local energy minimum of the input model. This is particularly useful in improving the quality of the input model, and in evaluating the sequence-backbone compatibility. The latter can be assessed from the magnitude of structural deviation compared with the input model and the reported average per-residue potential energy, which can be an orthogonal score to the Damietta energy values. Given the possible introduction of substantial backbone conformational change by this protocol, the ProteinMPNN-derived mutation probability matrix is recalculated, and is associated with the model if the user forwards it to other applications.

### Web-interface, output forwarding and usage scope

**Figure 3.**
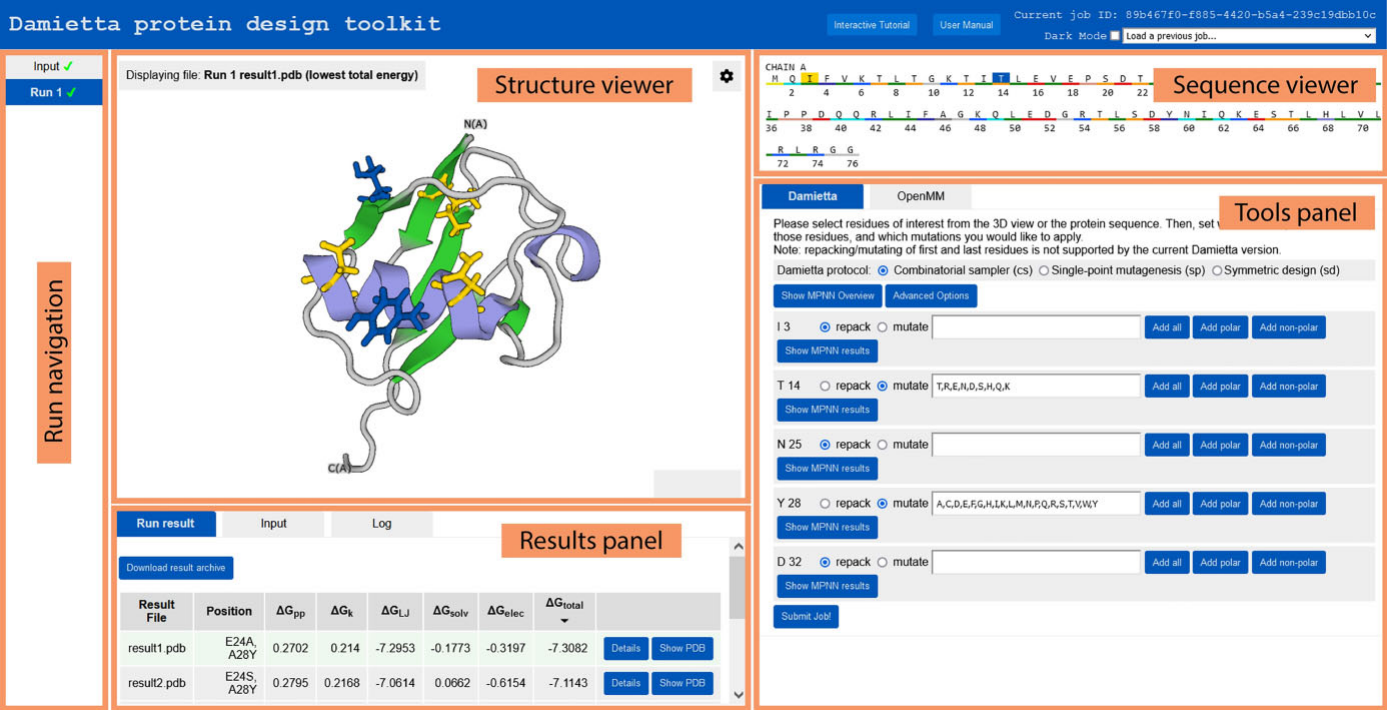
Overview of the Damietta Server web-interface. After the user uploads an input structure, various panels appear over web-interface as highlighted. The *run navigation* allows the user to toggle across running or completed jobs, or return to the input page to upload a new PDB. The *structure viewer* and *sequence viewer* display the 3D structure and amino acid sequence of input or output models. The *tools panel* allows the user to choose the tool, specify the input parameters and launch a run. Shown is an example of combinatorial sampler input with three repackable and two mutable positions. Finally, the results panel provides the user with the means of viewing and downloading the resulting models, sequences, and summary table. It also provides details on the job's input parameters and run log. Shown is an example of five output models, with the top model selected for viewing.

The user starts a workflow by uploading a protein structure file using the landing page. Structure models obtained from experimental data (e.g. Protein Data Bank (18)), predictions (e.g. AlphaFold (19)), or other sources are valid as input, which the user can upload as PDB or mmCIF file. Alternatively, the user can choose a previous run (from *run navigation* or the dropdown menu in the upper right corner), and select a model by toggling it from the *results panel* and clicking ‘show PDB’ (Figure 3). For newly uploaded files, the pre-processing of an input structure runs within seconds and a feed of the progress log is displayed to the user. After successful pre-processing, the *structure viewer*, *sequence viewer* and *tools panel* are activated, where the user can choose a tool. The two viewers allow the user to select the amino acid positions to be processed by Damietta tools. For an amino acid position, the user can ‘repack’ or ‘mutate’ an amino acid position. The mutation field has quick options for ‘all’, ‘polar’ or ‘non-polar’ amino acids. Additionally, ‘show MPNN results’ option provides the user with the log probability of each amino acid as predicted by ProteinMPNN (15).

The results panel lists the resulting models in a tabulated form, where the active model is selected by the ‘show PDB’ button. Each row displays the total energy and its broken-down values per energy term, which can be further expanded for per-residue values via the ‘details’ option. The active model can be chosen for further operations by specifying new run parameters from the tools panel. For applications where the user specifies more than a few mutations (i.e. ***cs*** and ***sd***), the user can specify advanced parameters such as the breadth (i.e. n_paths parameter) and depth (i.e. n_iters parameter) of sampling. It is thus recommended to raise these parameters for expanding the search scope, albeit at the cost of runtime. Executing multiple design runs, diversifying them (i.e. scramble_order randomization flag), and interlacing them with long minimization (***lm***) rounds until the energies cease to improve is also recommended. The server keeps track of the sequence changes for models forwarded through sequential cycles of designs, where the mutations from the initial input are displayed in the results table and the sequence viewer. The downloadable results archive contains the resulting design models, their FASTA sequences, and a summary table in CSV format.

Some factors and limitations must be considered by the user while using the server. Energy calculations in Damietta are computed from the point-of-view of a single side chain rotamer placed into the surrounding protein environment. Hence, all of the Damietta tools report the energy per-residue, averaged over all selected positions (either repacked or mutated). Thus, for valid comparisons between two proteins, equivalent (or all) positions must be selected. The presented combinatorial design tools (cs or sd) are deterministic, however, the input order of mutable amino acids is shuffled by default (Supplementary methods section), which constitutes the only source of stochasticity across runs. Therefore, it is recommended that the user launches several parallel runs with the same input, and considers any cross-run diversity in the resulting sequences. Additionally, the runtime of a combinatorial design protocol is greatly impacted by the number of mutable positions and the number of target mutations at each position. It is thus advisable that the user guides the search, for instance, by using polar/non-polar, or high-likelihood ProteinMPNN subsets. Finally, it is worth highlighting that the current version of the Damietta does not yet support non-proteinogenic chemotypes, such as small-molecule ligands, co-factors, or post-translational modifications. However, future upgrades will gradually cover additional chemotypes.

## Discussion

The sampling throughput, unbiased rotamer library, and first principles energy function distinguish the Damietta Server from the existing protein design servers that rely on evolutionary (6,20) or statistical scoring functions (21,22). Furthermore, the molecular dynamics-derived rotamer library deployed in Damietta has key advantages over widely-used Protein Data Bank-derived libraries. For instance, the former covers a much larger conformational space, and readily samples non-rotameric degrees of freedom as well as other conformational distortions affecting bond lengths or planarity (13,23). By relying on molecular mechanics, a rotamer library can be readily extended to cover non-standard amino acid chemotypes, under the same force field. Finally, the tensorized energy calculations in the Damietta Server enables fast sampling of a large number of rotamers. This is further leveraged by scalable computing cloud resources provided by the German Network for Bioinformatics Infrastructure for this project. Perhaps the most important advantage of the Damietta Server is seamlessly integrating different design and analysis tools into a single platform, which combined, are more useful than the sum of their parts. This architecture allowing the user to forward structures across different design tools was inspired by the Tübingen bioinformatics toolkit (24,25). Finally, the Damietta Server enables users to run up-to-date protein design tools, and easily visualize their results on the user-friendly graphical interface.

The versatile tools on the Damietta Server are useful for several applications. For instance, the combinatorial sampler can be used to design protein structures or protein-protein binding interfaces. Likewise, the symmetric design application can be used for the design of repeat proteins or homo-oligomeric assemblies, while imposing symmetric sequence constraints. This sequence-only symmetry allows for more exhaustive conformational sampling, and thus better scoring of symmetrized designs. Another example is using the single-point mutagenesis tool for mutagenic scanning to guide the creation of focused sequence libraries for *in vitro* display. Specifically, we have successfully used the Damietta single-point mutagenesis tool to guide the affinity maturation of designed modulators of a human cytokine receptor (granulocyte-colony stimulating factor receptor). This computationally-guided library yielded activators that were more potent *in vitro* and *in vivo*, when compared to the starting template (14). Alternatively, the single-point mutagenesis tool, separately or in combination with the long minimization protocol, can be useful to assess the impact of missense mutations on the structure, and subsequently the function, of a protein. This level of atomistic modeling of missense mutations is faster and better rationalized than if performed using deep learning methods (26). Finally, the repacking and long minimization protocols can also be useful for sampling alternate conformational states and assessing the energy of input structures. Here, the reporting of the decomposed energy terms could be useful, for example, in elaborating per-residue solvation free energy, which can be instructive in comparing solubility of different proteins (27).

Moving forward, our future development will aim to broaden the spectrum of applications available through the Damietta Server. Specifically, on the sampling side, we plan to incorporate protocols for multi-state design, local protein-protein docking, and tempering molecular dynamics routines. On the scoring side, we plan to enable setting up membrane environment. Moreover, we also plan to extend the number of chemotypes supported to encompass modified and tautomeric states of proteinogenic amino acids, non-standard amino acids, and metabolite molecules. Expanding this chemical space on the Damietta platform can be achieved through the combined use of LigandMPNN (28) for sequence predictions and CGenFF-derived parameters (29) for physical modeling.

## Supplementary Material

gkae297_Supplemental_File

## Data Availability

This web server is free and open to all users and there is no login requirement at https://damietta.de/.
