# Supplementary material for: The Damietta Server: a comprehensive protein design toolkit
Source: Nucleic Acids Res. 2024 Apr 25;52(W1):W200–6. doi: 10.1093/nar/gkae297 (PMC11223796; doi:10.1093/nar/gkae297)
Supplement: gkae297_Supplemental_File [file gkae297_supplemental_file.docx]

# *Supplementary Methods*

***Input preparation***

Pre-processing of the uploaded structures uses the PDBFixer module from OpenMM library (version 8.0) [1] to: i) substitute non-standard residues by their standard counterparts, ii) remove hetero atoms, and iii) add missing hydrogen or side chain heavy atoms. This is followed by aliasing atom names according to their CHARMM36 types, and uniquely renumbering all residue indices. Secondary structure annotation is then performed using the STRIDE software [2], and unconditional mutation probabilities matrices are computed in standard and solubility-enhancing modes of ProteinMPNN [3]. To fix any coordinates irregularities introduced during the addition of missing atoms, a quick minimization of L-BFGS 100 steps is performed with no bond constraints, using the OpenMM library.

***cs protocol***

The ***cs*** protocol in the current version (v1.60) follows a similar flow control as described before [4], with a minor upgrade to ensure the lowest-energy n_paths models represent unique sequences rather than unique conformations. The overall algorithm is implemented as follows:

cs

input: starting structure model

mutable positions

repackable positions

top_m

n_paths

n_iters

scramble_order

output: list of few lowest energy design models

start

// initialize

set few_list as empty list

if scramble_order is True:

set mutable_positions_list as shuffle_order(mutable positions)

set repackable_positions_list as repackable positions

append few_list with starting structure model

// search

for iter from n_iters to 0:

for each mutable_position in mutable_positions_list:

set many_list as empty list

for each min_model in few_list:

set mutants_list as generate_mutants(min_model, mutable_position)

for mutant_model in mutants_list:

local_score(mutant_model)

for mutant_model in mutants_list:

if mutant_model energy rank <= top_m:

append many_list with mutant_model

set combined_list to merge(few_list, many_list)

global_repack_and_score(combined_list)

set few_list as empty list

for model in combined_list:

if model energy rank <= n_paths:

append few_list with model

return few_list

end

The sampling parameters top_m, n_paths, and n_iters were set to 3, 5, and 1, respectively, as shown in the pseudocode. The scoring parameters for both repacking and mutagenesis tasks were set to the following values:

mut_max_lj = rpk_max_lj = 5

mut_w_pp = rpk_w_pp = 1

mut_w_k = rpk_w_k = 1

mut_w_lj = rpk_w_lj = 1

mut_w_solv = rpk_w_solv = 1

mut_w_elec = rpk_w_elec = 0.25

***sd protocol***

The ***sd*** protocol introduces sequence symmetry constraints to the tree swarm algorithm implemented in the cs protocol. The symmetric mutations are attempted in the input order, whereby the energy of the mutation is locally scored as the average of all of symmetrically-related residues. This also includes an added conditional that specifically eliminates the entire model if it contains at least one sterically-incompatible symmetric mutation (i.e. dG_lj > max_lj), as follows:

sd

input: starting structure model

mutable positions

repackable positions

symmetry constaints

top_m

n_paths

n_iters

scramble_order

output: list of few lowest energy design models

start

// initialize

set few_list as empty list

if scramble_order is True:

set mutable_positions_list as shuffle_order(mutable positions)

set repackable_positions_list as repackable positions

append few_list with starting structure model

// search

for iter from n_iters to 0:

for each mutable_position in mutable_positions_list:

set many_list as empty list

for each min_model in few_list:

set mutants_list as generate_mutants(min_model, mutable_position)

if symmetry constraint is specified

for mutant_model in mutants_list:

if symmetric positions associated with mutable_position:

generate_symmetric_mutations(mutant_model)

if a symmetric mutation causes a steric clash:

remove mutant_model from mutants_list

else

local_average_score(mutant_model)

else

local_score(mutant_model)

for mutant_model in mutants_list:

if mutant_model energy rank <= top_m:

append many_list with mutant_model

set combined_list to merge(few_list, many_list)

global_repack_and_score(combined_list)

set few_list as empty list

for model in combined_list:

if model energy rank <= n_paths:

append few_list with model

return few_list

end

The sampling and scoring parameters have the same values as the cs protocol.

***sp protocol***

The sp protocol generates only single-point mutant models. These models are initially created under the default max_lj = 5.0 kcal/mol, however, if they result in steric clashes, under an artificially elevated max_lj ceiling with a value of 1×10^10^ kcal/mol. In the latter scenario the overall score would be inaccurate, nonetheless, the models are still generated as they may be useful in rationalizing any conformational incompatibility.

sp

input: starting structure model

mutable positions

repackable positions

output: list of all single-point mutant models

start

// initialize

set mutants_list as empty list

set mutable_positions_list as mutable positions

set repackable_positions_list as repackable positions

// generate

for each mutable_position in mutable_positions_list:

append mutants_list with generate_single_mutants(input_model, mutable_position)

for each mutant_model in mutants_list:

if mutation causes a steric clash:

enforce mutation with raised max_lj ceiling

global_repack_and_score(mutants_list)

return mutants_list

end

***lm protocol***

The lm tool performs up to 10,000 local L-BFGS minimization steps with the CHARMM36 parameters force field as deployed in OpenMM. For speed of execution, the simulation is performed with no solvent modeling, a nonbonded cutoff of 10 Å, and all-bond constraints. The resulting potential energy values are normalized by the total number of residues in the model to yield comparable per-residue values.

***Server architecture***

The Damietta protein design toolkit webserver employs a modular architecture comprised of a frontend node which serves the website and receives user inputs, and several worker nodes which process individual tool runs. The frontend is based on the Apache Web Server (Version 2.4) with the Flask Python web framework to handle user input. The website uses the pv JavaScript WebGL protein viewer [5] for displaying protein structures.

Job handling is done by the Python RQ module, a Redis based queuing system that schedules incoming computation jobs for processing in the background by the worker nodes. The server and network infrastructure for the Damietta protein design toolkit is provided by the cloud services of deNBI – German Network for Bioinformatics Infrastructure (<https://www.denbi.de/cloud>).

***References***

1. Eastman, P., et al., *OpenMM 7: Rapid development of high performance algorithms for molecular dynamics.* PLOS Computational Biology, 2017. **13**(7): p. e1005659 DOI: 10.1371/journal.pcbi.1005659.

2. Heinig, M. and D. Frishman, *STRIDE: a web server for secondary structure assignment from known atomic coordinates of proteins.* Nucleic Acids Res, 2004. **32**(Web Server issue): p. W500-2 DOI: 10.1093/nar/gkh429.

3. Dauparas, J., et al., *Robust deep learning–based protein sequence design using ProteinMPNN.* Science, 2022. **378**(6615): p. 49-56 DOI: 10.1126/science.add2187.

4. Maksymenko, K., et al., *The design of functional proteins using tensorized energy calculations.* Cell Reports Methods, 2023. **3**(8): p. 100560 DOI: <https://doi.org/10.1016/j.crmeth.2023.100560>.

5. Biasini, M., *pv: v1.8.1*. 2015, Zenodo.
